# Differences of Circulating CD25^hi^ Bregs and Their Correlations with CD4 Effector and Regulatory T Cells in Autoantibody-Positive T1D Compared with Age-Matched Healthy Individuals

**DOI:** 10.1155/2022/2269237

**Published:** 2022-01-17

**Authors:** Jie Zhang, Qi Fu, Yunqiang He, Hui Lv, Yu Qian, YuYue Zhang, Heng Chen, Xinyu Xu, Tao Yang, Kuanfeng Xu

**Affiliations:** Department of Endocrinology and Metabolism, The First Affiliated Hospital of Nanjing Medical University, Nanjing, China 210029

## Abstract

Circulating CD25^hi^ B cells, a subset of regulatory B cells in humans, are closely related to inflammation and autoimmune diseases. This study is aimed at investigating the alternation of CD25^hi^ Bregs and their correlation with CD4 effector and regulatory T cells in T1D individuals. We included 68 autoantibody-positive T1D and 68 age-matched healthy individuals with peripheral blood mononuclear cells (PBMCs) and assessed them with CD25^hi^ Bregs and CD4 effector or regulatory T cells by flow cytometry. Here, we demonstrate that the frequency of CD25^hi^ Bregs was significantly decreased in T1D subjects (*P* = 0.0016), but they were not affected by disease status (age at T1D diagnosis or duration) or T1D risk loci (rs2104286 or rs12251307) in *IL2RA* (all *P* > 0.05). Moreover, higher IgD (*P* = 0.043) and lower CD27 (*P* = 0.0003) expression was observed in CD25^hi^ Bregs of T1D individuals, but not the expression of IgM, CD24, or CD38 (all *P* > 0.05). Although there was no correlation between CD25^hi^ Bregs and CD4 effector T cell subsets in either T1D or healthy individuals (all *P* > 0.05), we found a positive correlation between CD25^hi^ Bregs and CD4 Tregs in healthy controls (Sp. *r* = 0.3544, *P* = 0.0249), which disappeared in T1D subjects (Sp. *r* = 0.137, *P* = 0.401). In conclusion, our results suggest that decreased CD25^hi^ Bregs and alternation of their phenotypes are features of T1D regardless of disease duration and T1D genetic risk loci, and an impaired balance between CD25^hi^ Bregs and CD4 Tregs might contribute to the pathogenesis of T1D.

## 1. Introduction

Type 1 diabetes (T1D) is an organ-specific autoimmune disease mediated by T cells against pancreatic *β* cells. The decreased number and impaired function of Tregs in T1D individuals result in an imbalance between Tregs and effector T cells and abnormal immune responses, which leads to the occurrence and progression of T1D [1, 2]. T cells, especially CD4 and CD8 conventional T cells with specificity for islet autoantigens [3], are critical in mediating the destruction of *β* cells. But B cells also play an essential role in the autoimmune destruction of *β* cells [4, 5], which mainly participate in the T cell immune response by producing autoantibodies, presenting antigens, secreting cytokines, and providing costimulatory signals [6–8].

Regulatory B cells (Bregs) are B lymphocytes that function by skewing T cell differentiation in favor of a regulatory phenotype in both mice and humans. According to surface markers, Bregs can be divided into different regulatory subsets, including B10 cells, plasmablasts, Br1 cells, and immature B cells in humans [9]. They are involved in the immune process by producing interleukin- (IL-) 10, IL-35, and transforming growth factor-*β* (TGF-*β*), inhibiting the proliferation of CD4 effector T cells, and enhancing the expression of FOXP3 and CTLA-4 in Tregs [10]. Studies have shown these Bregs are involved in the pathogenesis of T1D to some extent [11–14].

CD25 (also named as interleukin-2 *α*-chain receptor (IL-2RA)) is highly expressed in CD4 Tregs [15, 16], which is vital for Treg function [17] and the pathogenesis of many autoimmune diseases [2]. Studies also reveal that the CD19^+^CD25^+^ B cells (CD25^hi^ Bregs) are the first subtype of regulatory B cells in humans. These Bregs are partially similar to CD4 Tregs as they express significantly higher levels of the immunosuppressive cytokine IL-10 [10]. However, the alternation of these CD25^hi^ Bregs in T1D is still unclear.

Therefore, this study focused on the alternation of circulating CD25^hi^ Bregs in T1D subjects and the effect of disease status, as well as T1D risk loci in *IL-2RA*, on the frequency of CD25^hi^ Bregs. Furthermore, we also assessed their correlations with CD4 effector and regulatory T cells in both T1D and healthy donors.

## 2. Materials and Methods

### 2.1. Subjects

This study included 68 T1D subjects from the Department of Endocrinology, the First Affiliated Hospital of Nanjing Medical University. The diagnosis of T1D met the WHO criteria, and T1D subjects had at least one positive islet-specific autoantibody, including zinc transporter-8 autoantibody (ZnT8A), glutamate decarboxylase autoantibody (GADA), and insulinoma-related-2 autoantibody (IA-2A). ZnT8A, GADA, and IA-2A were measured by radio-binding assays described previously [18]. Sixty-eight age-matched healthy controls were from the same geographic area and had no diabetes or other autoimmune diseases. All samples were collected after all participants and/or their guardians had written informed consent. This study was approved by the Ethics Committee of the First Affiliated Hospital of Nanjing Medical University and was conducted in accordance with the principles of the Declaration of Helsinki.

### 2.2. Cell Staining and Multicolor Flow Cytometry

Ficoll density gradient centrifugation was used to separate human peripheral blood mononuclear cells (PBMCs) at study entry and frozen at a core facility. Thawed PBMCs were stained with aqua for live/dead cells; for CD25^hi^ Bregs panel, these cells were stained with CD19 (HIB19), CD25 (M-A251), IgM (MHM-88), IgD (IA6-2), CD24 (ML5), CD27 (323), CD38 (HIT2), and dump (CD3/CD14/CD56/Aqua); for T effector and regulatory cells, they were stained with CD3 (SK7), CD4 (SK3), CD8 (SK1), CD25 (M-A251), CD45RA (HI100), CCR7 (GO43H7), FOXP3 (259D/C7), and CTLA-4 (BNI3), as previously described [18]. PBMCs are run on FACSAria II (BD Biosciences) and analyzed by FlowJo v10 software.

### 2.3. Genotyping

DNeasy blood and tissue kit (Qiagen) was used to extract genomic DNA from isolated PBMCs. Genome-wide association studies (GWAS) revealed T1D-related risk loci in/nearby *IL2-RA*, including rs2104286 and rs12251307 (from http://www.t1dbase.org). PCR was performed on ABI 7900HT by the TaqMan method to assess these loci.

### 2.4. Statistical Analysis

The Mann–Whitney unpaired *t*-test evaluated the comparison between the two groups. Comparisons of immune phenotypes between CD25^hi^ Bregs and CD25^−^ B cells from the same individual were performed using a paired two-tailed Student's *t*-test. The Spearman rank test determined the correlations between variables. All statistical data were analyzed using GraphPad Prism 7.0 (GraphPad Software, La Jolla, California). A *P* value of <0.05 was considered statistically significant.

## 3. Results

### 3.1. The Frequency of CD25^hi^ Bregs Decreases Significantly in T1D Individuals

**Figure 1:**
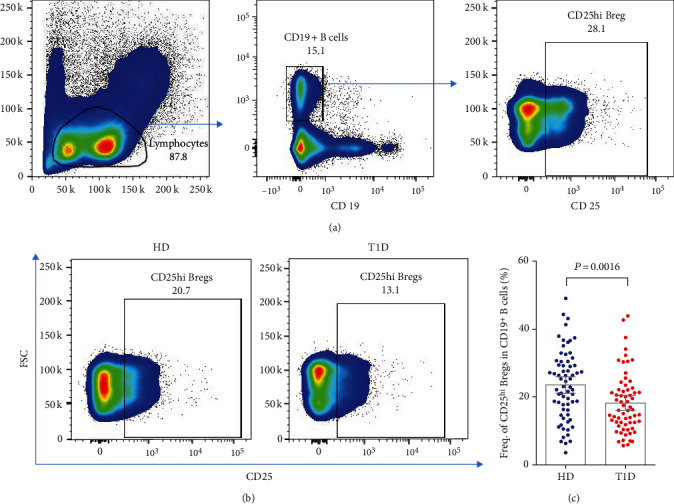
Differences in circulating CD25^hi^ Bregs between autoantibody-positive T1D and age-matched healthy individuals. (a, b) Representative dot plots for gating CD25^hi^ Bregs in healthy donor and T1D subject. (c) Evaluation of the frequency of CD25^hi^ Bregs in CD19^+^ B cells between T1D and healthy controls. A *P* value below 0.05 indicates a significant difference between groups.

The clinical characteristics of T1D and healthy donors are shown in Table S1, matched for age and gender between the two groups. Representative dot plots gating CD25^hi^ Bregs in T1D and healthy donors are shown in Figures 1(a) and 1(b). Our results indicate that age at the time of blood donation does not affect the frequency of CD25^hi^ Bregs in T1D or healthy controls (Figures S1A and B), but they significantly decrease in T1D compared with age-matched healthy individuals (23.5% ± 1.2 vs. 18.2% ± 1.1, *P* = 0.0016), as shown in Figure 1(c).

### 3.2. CD25^hi^ Bregs Do Not Correlate with Age at T1D Diagnosis, T1D Duration, or T1D Risk Loci in the *IL2RA* Region

**Figure 2:**
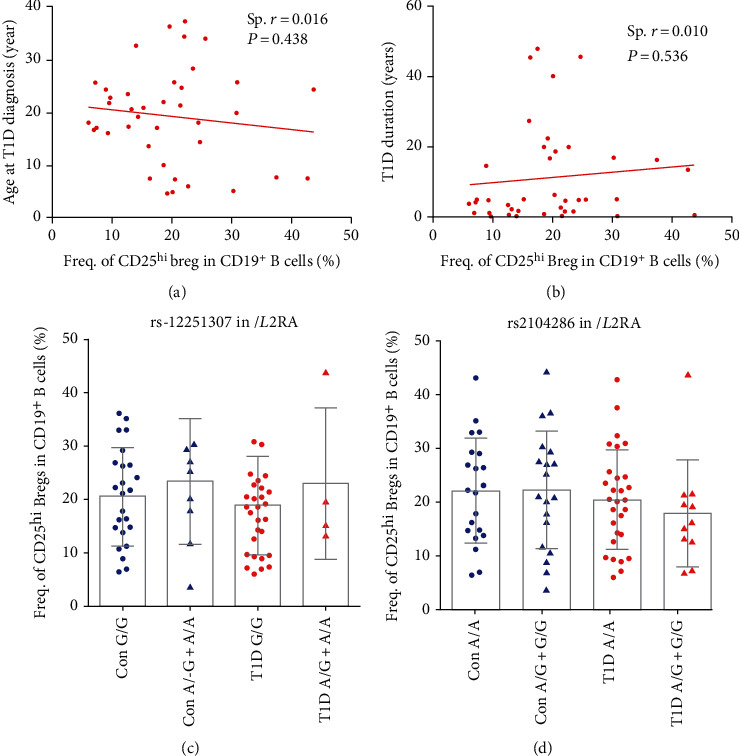
The effect of disease status and T1D risk loci (rs12251307 and rs2104286) in IL2RA on CD25 expression in CD19^+^ B cells. Correlation between the frequency of CD25^hi^ Bregs in CD19^+^ B cells and age at (a) T1D diagnosis and (b) T1D duration in T1D subjects. The correlation was determined by the Spearman rank test. (c, d) Con represents healthy controls. For both healthy controls and T1D individuals, comparisons between wild genotype and homozygote+ heterozygote and were performed by unpaired *t*-test with Welch's correction. A total of 40 T1D individuals and 40 healthy controls were enrolled for the analysis. A *P* value below 0.05 indicates a significant difference for different genotypes in each group.

Diseases status and genetic risk loci may contribute to the frequency of immune cell subsets. Our results demonstrate that the frequency of CD25^hi^ Bregs does not correlate with age at T1D diagnosis or duration (Figures 2(a) and 2(b)), suggesting they might not be affected by disease status. Besides, GWAS have revealed several T1D genetic loci in the *IL2RA* region, including rs11594656, rs12251307, rs12722495, and rs2104286. But only rs2104286 and rs12251307 are common variants in the Chinese Han population. We further assessed their contribution to the frequency of CD25^hi^ Bregs. Although phenoscanner database (http://www.phenoscanner.medschl.cam.ac.uk/) indicated that rs2104286 had an eQTL effect on *IL2RA* in esophagus gastroesophageal junction and heart atrial appendage (from GTEx.v7) and rs12251307 had an eQTL effect on *IL2RA* in whole blood (from BIOSQTL), they are not associated with CD25 expression in CD19^+^ B cells in either healthy controls or T1D individuals (all *P* > 0.05), as shown in Figures 2(c) and 2(d).

### 3.3. Higher IgD and Lower CD27 Expression in CD25^hi^ Bregs Is Observed in T1D Individuals

We next performed a comparative phenotypic analysis for CD25^hi^ Bregs and CD25^−^ B cells by evaluating the common surface marker, including IgM, IgD, CD24, CD27, and CD38. We observe significantly higher IgD and CD38 expression and lower CD24 and CD27 expression in CD25^hi^ Bregs compared to CD25^−^ B cells in healthy individuals (all *P* < 0.0001) (Figure S2A). The results are similar for the alternation of IgD, CD27, and CD38 expression in CD25^hi^ Bregs compared to CD25^−^ B cells in T1D individuals (all *P* < 0.0001) (Figure S2B). These results suggest CD25^hi^ Bregs are a specific distinct subpopulation.

**Figure 3:**
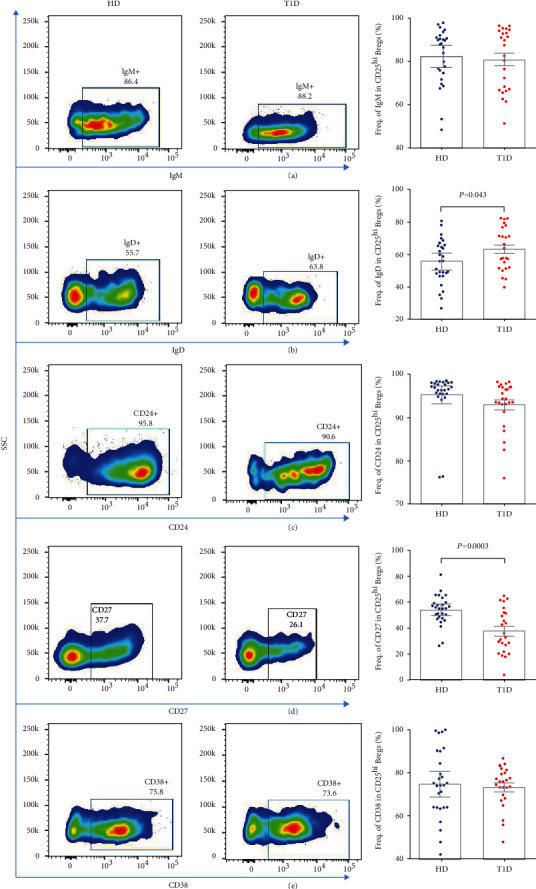
Differences of (a) IgM, (b) IgD, (c) CD24, (d) CD27, and (e) CD38 expression in circulating CD25^hi^ Bregs between autoantibody-positive T1D and healthy individuals. A *P* value below 0.05 indicates a significant difference between groups.

Continuing our analysis, IgD expression in CD25^hi^ Bregs increases, while CD27 expression decreases significantly in T1D individuals (*P* = 0.043 and 0.0003, respectively), but IgM, CD24, and CD38 expression does not alter, as shown in Figures 3(a)–3(e). Although IgM, IgD, CD24, and CD38 expression does not correlate with age at the time of donation, our results show that the expression of CD27 in CD25^hi^ Bregs has a positive correlation with age at drawn in healthy donors (Figures S3A–E). These suggest age-matched individuals are essential for the comparisons. The expression of CD27 in CD25^hi^ Bregs also reduces significantly in T1D subjects compared with age-matched healthy donors (*P* = 0.0157), as shown in Figure S4A. However, the expression of CD27 in CD25^hi^ Bregs does not correlate with either age at T1D diagnosis or duration (Figures S4B and C).

### 3.4. Significant Correlation between CD25^hi^ Bregs and CD4 Tregs in Healthy Donors Disappears in T1D Individuals

**Figure 4:**
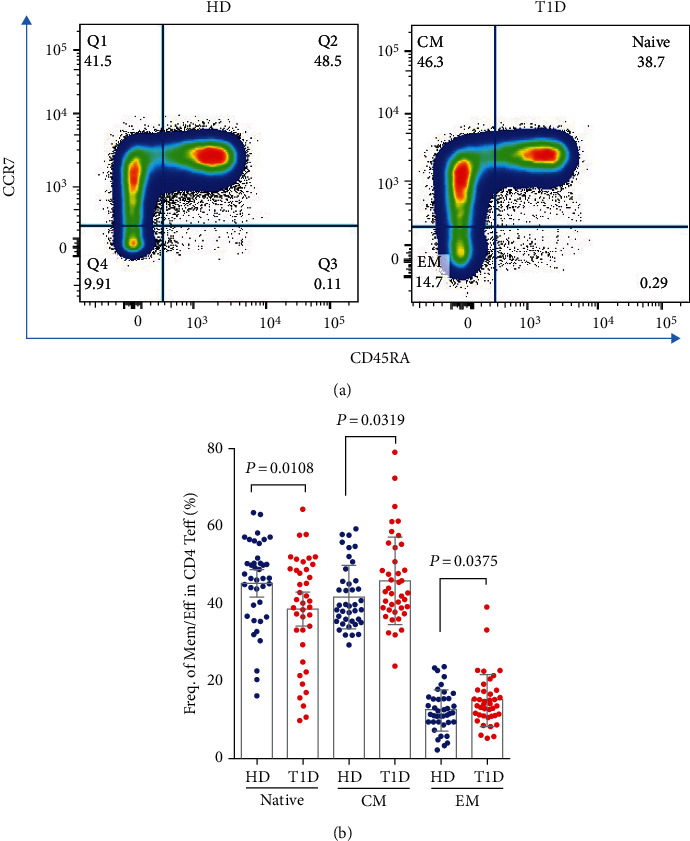
Differences in circulating naïve/CM/EM subsets in total CD4 effector T cells between autoantibody-positive T1D and age-matched healthy individuals. (a) Representative dot plots for gating naïve/CM/EM subsets in total CD4 effector T cells in a healthy donor (HD) and T1D subject. (b) Evaluation of circulating naïve/CM/EM subsets in total CD4 effector T cells between T1D and healthy individual controls. A *P* value below 0.05 indicates a significant difference between groups.

Our results show that neither the frequency [19] nor the number (Figures S5A–C) of CD4 Tregs alters in T1D subjects. Here, we evaluated the differences of CD4 effector T cell subsets between T1D and age-matched healthy individuals. Although the frequency of total CD4 effector T cells in total T cells shows no difference (Figure S6), T1D individuals have lower frequency of naïve CD4 T cells and higher frequency of central memory (CM) and effect memory (EM) CD4 T cells in both CD4 effector (Figures 4(a) and 4(b)) and CD3 T (Figure S5) cells.

**Figure 5:**
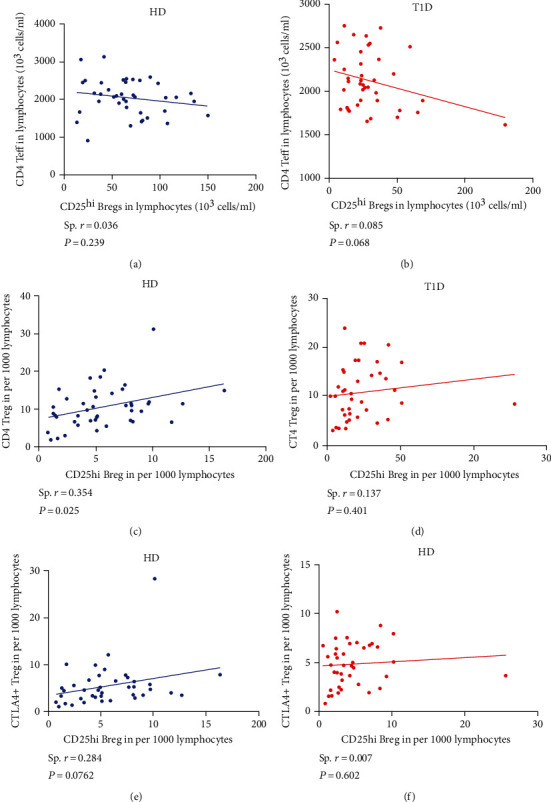
Correlations between CD25^hi^ Bregs and effector CD4 T cells, CD4 Tregs, and CTLA-4^+^ Tregs in healthy control and T1D individuals. Correlation analysis between CD25^hi^ Bregs and effector CD4 T cells from (a) healthy donors (HD) and (b) T1D individuals. Correlation analysis between CD25^hi^ Bregs and CD4 Tregs from (c) HD and (d) T1D individuals. Correlation analysis between CD25^hi^ Bregs and CTLA-4^+^ Tregs from (e) HD and (f) T1D individuals. Spearman correlations were performed for these correlations. A *P* value < 0.05 was considered significant.

Furthermore, our previous study also demonstrated that CD4 Tregs were significantly correlated with regulatory monocytes in healthy controls, which disappeared in T1D individuals [18]. Here, we further assessed the correlation between CD25^hi^ Bregs and CD4 effector and regulatory T cells. As shown in Figures 5(a) and 5(b) and Figures S7A–F, no correlation between CD25^hi^ Bregs and CD4 effector T cell subsets is observed in either T1D or healthy donors (all *P* > 0.05). As shown in Figures 5(c) and 5(d), we observe a positive correlation between CD25^hi^ Bregs and Tregs in healthy controls (Spearman *r* = 0.354, *P* = 0.025), which disrupted in T1D individuals (Spearman *r* = 0.137, *P* = 0.401). In addition, CD25^hi^ Bregs tend to correlate with CTLA-4^+^ Tregs in healthy controls (Spearman *r* = 0.284, *P* = 0.076), but not T1D individuals (Spearman *r* = 0.007, *P* = 0.602), as shown in Figures 5(e) and 5(f).

## 4. Discussion

Studies have demonstrated different Breg subsets in both mice and humans [9]. In mice, studies showed that Bregs could prevent or delay autoimmune diabetes in nonobese diabetic (NOD) mice. Tian et al. initially explored the role of Bregs in T1D in nonobese diabetic (NOD) mice [11, 12]. However, the conclusions are not entirely consistent in humans. Thompson et al. found that the secretion of IL-10 from circulating Bregs in T1D subjects was not statistically significant compared with healthy controls [13]. El-Mokhtar et al. found that Breg subgroups CD24^hi^CD27^+^ (B10) and CD24^hi^CD38^hi^ decreased significantly in T1D subjects, which were negatively correlated with fasting blood glucose and glycosylated hemoglobin [14].

CD25^hi^ Bregs, one of the regulatory B cells in humans [17], are closely related to inflammation, malignant tumors, and autoimmune diseases. Hjalmar et al. found that the average proportion of CD19^+^ B cells expressing CD25 in subjects with chronic lymphocytic leukemia was significantly higher than that in healthy controls, and the median treatment time of these patients was shorter than that of patients with CD25^−^ B cells [20]. de Andrés et al. found that CD25^hi^ Bregs increased significantly in the cerebrospinal fluid compared with peripheral blood. Moreover, these Bregs are higher in multiple sclerosis patients with relapsed symptoms than nonclinically active multiple sclerosis patients [21]. Another study showed that higher CD25^hi^ Bregs are independently associated with better graft function in renal transplant recipients [22]. Our study found that the frequency of CD25^hi^ Bregs decreased significantly in T1D subjects, which is another evidence of their effect on autoimmune diseases. In addition, although studies demonstrate that the development of B lymphocytes and changes in receptor diversity are affected by the aging process [23], we did not find any correlation between CD25^hi^ Bregs and the age at drawn in either T1D or healthy individuals.

Besides, disease status and genetic risk loci may also affect these Bregs. We did not find any correlation between CD25^hi^ Bregs and disease onset and duration. As for multiple genetic risk loci in/near *IL2RA*, they were reported to affect CD25 expression in whole blood and other tissues and help reduce the frequency of IL-2R signaling in T1D and MS patients [24]. But we did not find any effect of these loci on CD25 expression on CD25^hi^ Bregs, likely due to lower surface expression. These suggested these risk loci might affect CD25 expression in a cell type-specific manner.

Furthermore, our results indicated that compared to CD25^−^ B cells, CD25^hi^ Bregs had a distinct phenotype in higher expression of CD24 and CD27, meanwhile lower expression of IgD and CD38. IgD participates in the initiation of B cell production of antibodies, attenuates the survival of mature B cells, and participates in inhibiting nonspecific B cell activation and autoimmunity [25]. Our study revealed higher IgD expression in CD25^hi^ Bregs in T1D, which suggested higher autoimmune response in T1D status. CD27 is a regulator of B cell activation and antibody production [26]. Our study found that the expression of CD27 in CD25^hi^ Bregs significantly decreased in T1D subjects. Interestingly, CD27 expression was positively associated with age at drawn in both T1D and healthy individuals. These suggest age-matched individuals are essential for comparing immune cells between T1D subjects and healthy donors, and CD27 may have a particular influence on the production and immune function of CD25^hi^ Bregs.

Furthermore, studies have shown that CD25^hi^ Bregs could increase CD4 Tregs while reducing Th17 cells [27]. Kessel et al. found that human CD25^hi^ Bregs inhibited the proliferation of CD4 T cells and enhanced the expression of Foxp3 and CTLA-4 in Tregs [10]. Another study also indicated CD25^hi^ Bregs that secrete IL-10 are a subgroup of cells with different functions that affect the fate of T cells in patients with leprosy. These cells convert effector T cells into Treg and enhance Treg activity [28]. Our study found that CD4 Tregs positively correlated with CD25^hi^ Bregs in healthy individuals were disrupted in T1D subjects. Based on these studies, we speculated that the suppressive function of CD25^hi^ Bregs might be diminished in T1D individuals, partially due to the decreased IL-10 secretion in CD25^hi^ Bregs, which deserves further exploration with extra more studies.

Our study also has some limitations. Firstly, we only found a tendency of correlation between CD25^hi^ Bregs and CTLA-4^+^ Tregs in healthy individuals. It should be further investigated with more sample size to assess the bona fide correlation. Secondly, the phenotype of CD25^hi^ Bregs should also need further confirmation by other independent studies. Thirdly, the functional cytokines of CD25^hi^ Bregs, including IL-10, IL-35, and TGF-*β*, should be evaluated in T1D and age-matched healthy controls by further studies.

In conclusion, this study found decreased circulating CD25^hi^ Bregs and altered phenotype in CD25^hi^ Bregs T1D individuals, and the positive correlation between CD25^hi^ Bregs and Tregs in healthy donors was disrupted in T1D subjects. CD25^hi^ Bregs might contribute to the onset and development of T1D, but the related mechanism remains to be further studied.

## Data Availability

We have provided our data in the Supplementary Information files that we have submitted alongside our manuscript.

## Supplementary Materials

Supplementary Materials Figure S1: correlations between CD25^hi^ Bregs in CD19^+^ B cells and age at drawn in both healthy donor and T1D subjects. HD represents healthy controls. A *P* value below 0.05 indicates a significant correlation. Figure S2: differences in IgM, IgD, CD24, CD27, and CD38 expression in circulating CD25^hi^ Bregs compared to CD25^−^ B cells in healthy donors (HD) (A) and T1D subjects (B). Comparisons between T1D and healthy controls were performed by paired *t*-test with Wilcoxon matched-pairs signed rank test. A *P* value < 0.05 was considered as significant. Figure S3: correlations between frequency of phenotype expression in CD25^hi^ Bregs and age at drawn in healthy donors. A–E represent IgM, IgD, CD24, CD27, and CD38. A *P* value below 0.05 indicates a significant correlation. Figure S4: differences in CD27 expression (A) in circulating CD25^hi^ Bregs between autoantibody-positive T1D and age-matched healthy individuals and the correlation with disease status (B, age at T1D diagnosis; C, T1D duration). A *P* value < 0.05 was considered as significant. Figure S5: evaluation of the number of CD4 Tregs in per 1000 lymphocytes (A), CD3 T cells (B), and CD4 T cell subsets (C) between T1D and healthy controls. Figure S6: differences in circulating CD4 effector T cell subsets in CD3 T cells between autoantibody-positive T1D and age-matched healthy individuals. A *P* value below 0.05 indicates a significant difference between groups. Figure S7: correlations between CD25^hi^ Bregs and CD4 effector T cell subsets in healthy donors (A–C) or T1D individuals (D–F). Frequency of CD4 effector T cell subsets in CD3 T cells (A) and CD4 T cells (B). CM: central memory; EM: effector memory. A *P* value below 0.05 indicates a significant correlation. Table S1: clinical features of the included T1D and healthy donors.
