# Supplementary material for: Differences of Circulating CD25hi Bregs and Their Correlations with CD4 Effector and Regulatory T Cells in Autoantibody-Positive T1D Compared with Age-Matched Healthy Individuals
Source: J Immunol Res. 2022 Jan 17;2022:2269237. doi: 10.1155/2022/2269237 (PMC8786465; doi:10.1155/2022/2269237)
Supplement: Supplementary Materials — Figure S1: correlations between CD25hi Bregs in CD19+ B cells and age at drawn in both healthy donor and T1D subjects. HD represents healthy controls. A P value below 0.05 indicates a significant correlation. Figure S2: differences in IgM, IgD, CD24, CD27, and CD38 expression in circulating CD25hi Bregs compared to CD25− B cells in healthy donors (HD) (A) and T1D subjects (B). Comparisons between T1D and healthy controls were performed by paired t-test with Wilcoxon matched-pairs signed rank test. A P value < 0.05 was considered as significant. Figure S3: correlations between frequency of phenotype expression in CD25hi Bregs and age at drawn in healthy donors. A–E represent IgM, IgD, CD24, CD27, and CD38. A P value below 0.05 indicates a significant correlation. Figure S4: differences in CD27 expression (A) in circulating CD25hi Bregs between autoantibody-positive T1D and age-matched healthy individuals and the correlation with disease status (B, age at T1D diagnosis; C, T1D duration). A P value < 0.05 was considered as significant. Figure S5: evaluation of the number of CD4 Tregs in per 1000 lymphocytes (A), CD3 T cells (B), and CD4 T cell subsets (C) between T1D and healthy controls. Figure S6: differences in circulating CD4 effector T cell subsets in CD3 T cells between autoantibody-positive T1D and age-matched healthy individuals. A P value below 0.05 indicates a significant difference between groups. Figure S7: correlations between CD25hi Bregs and CD4 effector T cell subsets in healthy donors (A–C) or T1D individuals (D–F). Frequency of CD4 effector T cell subsets in CD3 T cells (A) and CD4 T cells (B). CM: central memory; EM: effector memory. A P value below 0.05 indicates a significant correlation. Table S1: clinical features of the included T1D and healthy donors. [file 2269237.f1.doc]

**Table S1 Clinical features of the included T1D and healthy donor**s

|  | **T1D** | **HD** |
| --- | --- | --- |
| n | 68 | 68 |
| Gender (Male /Female) | 29/39 | 29/39 |
| Age (Years) | 30.4±11.3 | 30.8±11.6 |
| BMI (kg/m2) | 20.4±1.6 | 22.6±2.1 |
| Fasting blood glucose level (mmol/L) | 12.5±3.9 | 5.1±0.3 |
| HbA1c (%) | 7.9±1.8 | 4.8±0.4 |
| Age of onset (Years) | 19.5±8.9 | NA |
| Disease duration (Years) | 4.8 (1.5-16.8) | NA |
| ZnT8A, n (%) | 26 (38.2) | NA |
| GADA, n (%) | 40 (58.8) | NA |
| IA-2A, n (%) | 23 (33.8) | NA |

Note: HD, Healthy control donors, ZnT8A: zinc transporter-8 autoantibody; GADA: glutamate decarboxylase antibody; IA-2A: insulinoma-related-2 autoantibody; IAA: insulin antibody; -: no data; age at onset mean age ± Standard deviation; Median duration of type 1 diabetes (quartile).


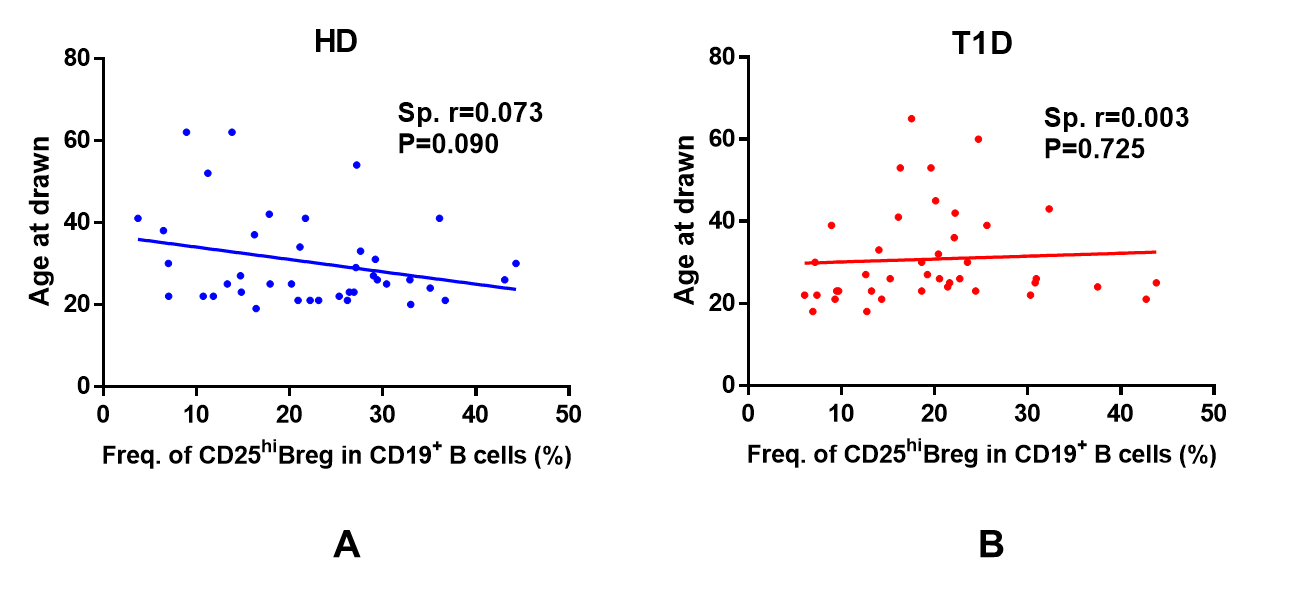


**Figure S1. Correlations between CD25^hi^ Bregs in CD19^+^ B cells and age at drawn in both healthy donor and T1D subjects.** HD represents healthy controls. A *P* value below 0.05 indicates a significant correlation.


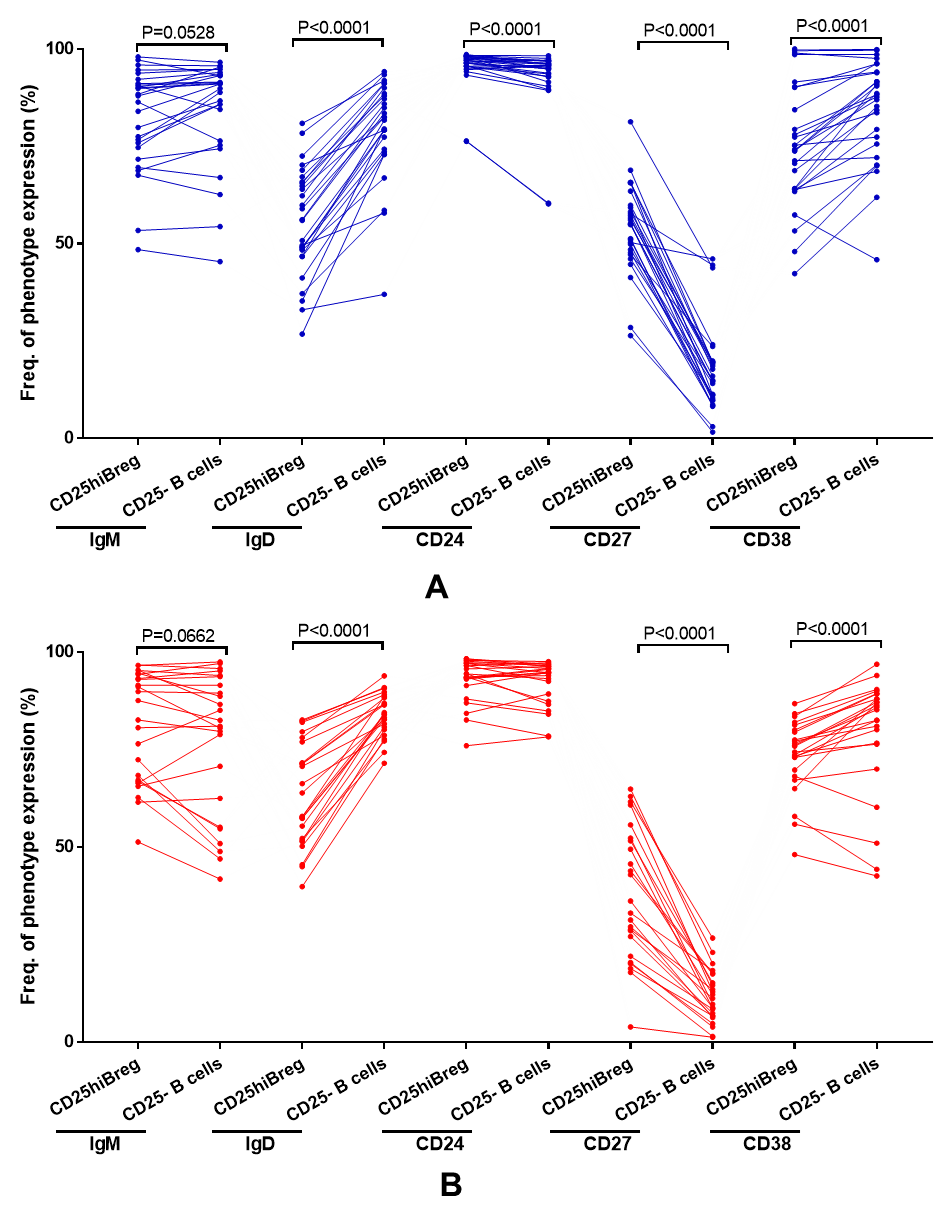


**Figure S2. Differences in IgM, IgD, CD24, CD27 and CD38 expression in circulating CD25^hi^ Bregs compared to CD25^-^ B cells in healthy donors (HD) (A) and T1D subjects (B).** Comparisons between T1D and healthy controls were performed by paired t test with Wilcoxon matched-pairs signed rank test. A *P* value <0.05 was considered as significant.


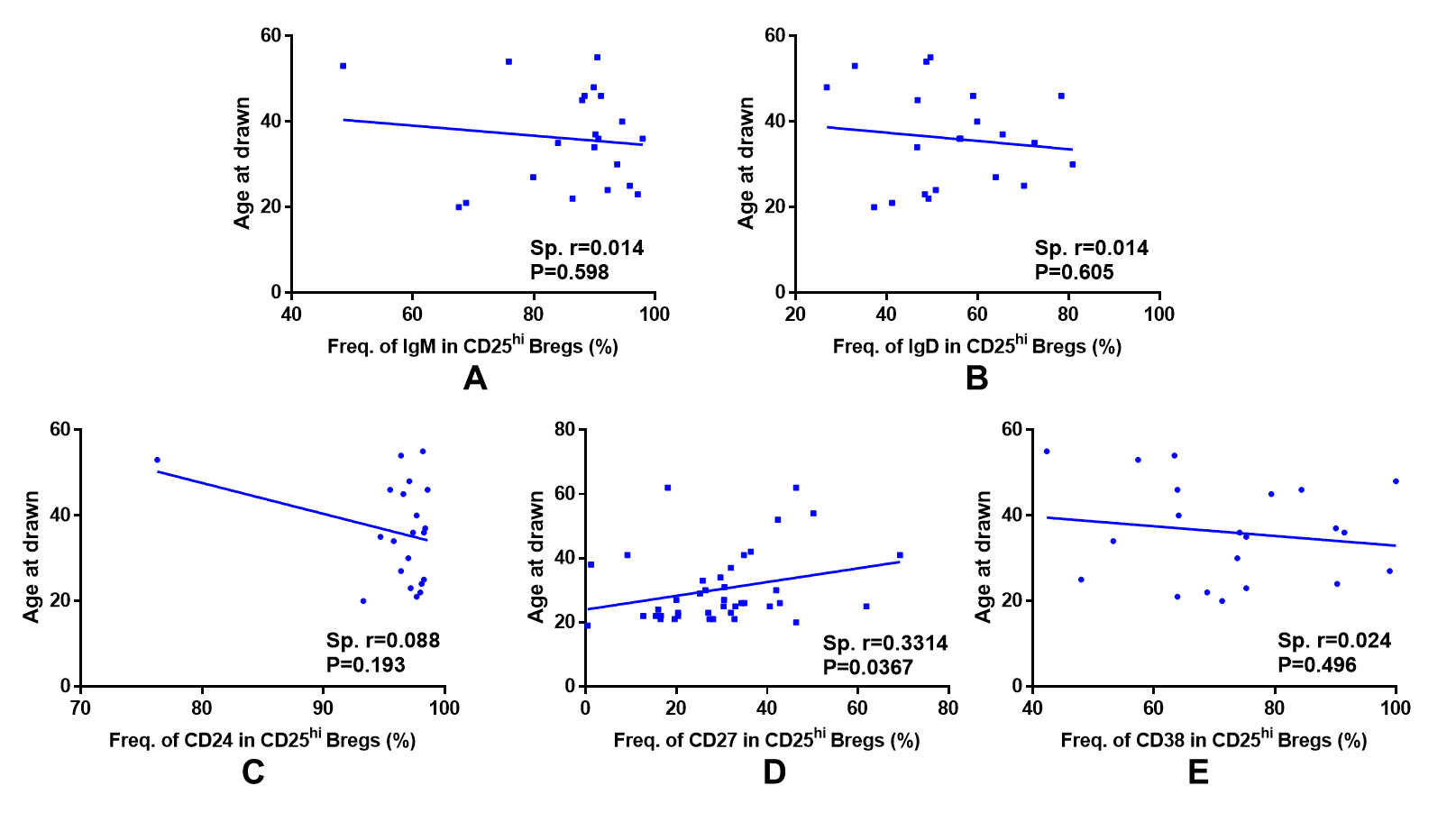


**Figure S3. Correlations between frequency of phenotype expression in CD25^hi^ Bregs and age at drawn in healthy donors. A-E** represent IgM, IgD, CD24, CD27 and CD38. A *P* value below 0.05 indicates a significant correlation.


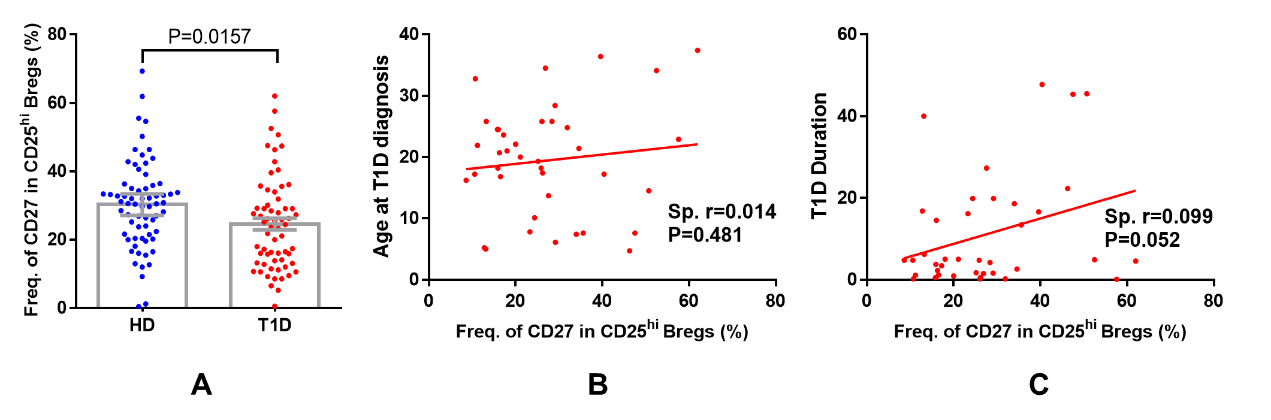


**Figure S4. Differences in CD27 expression (A) in circulating CD25^hi^ Bregs between autoantibody-positive T1D and age-matched healthy individuals and the correlation with disease status (B, age at T1D diagnosis; C, T1D duration).** A *P* value <0.05 was considered as significant.

**Figure S5.** Evaluation of the number of CD4 Tregs in per 1000 lymphocytes (A), CD3 T cells (B) and CD4 T cell subsets (C) between T1D and healthy controls.

**Figure S6. Differences in circulating CD4 effector T cell subsets in CD3 T cells between autoantibody-positive T1D and age-matched healthy individuals.** A P value below 0.05 indicates a significant difference between groups.


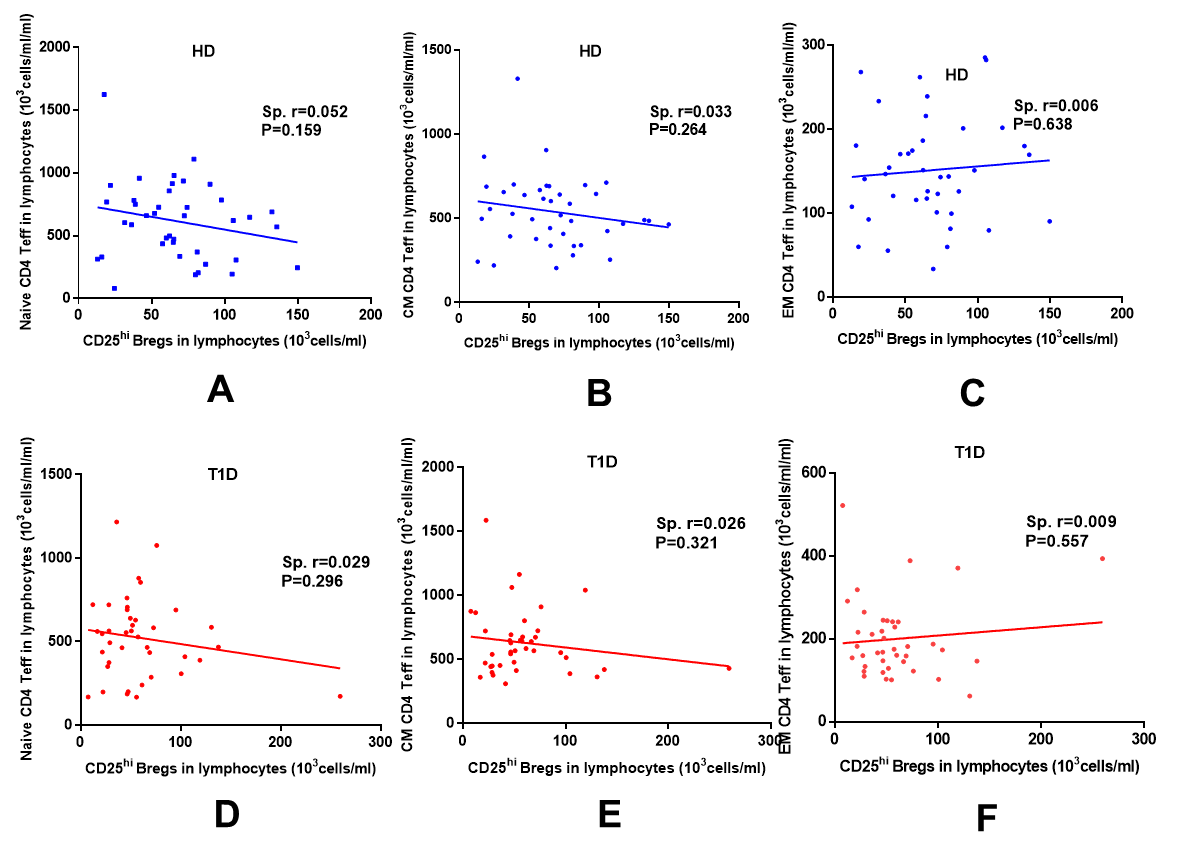


**Figure S7. Correlations between CD25^hi^ Bregs and CD4 effector T cell subsets in healthy donors (A-C) or T1D individuals (D-F). A,** Frequency of CD4 effector T cell subsets in CD3 T cells (**A**) and CD4 T cells (**B**). CM, central memory; EM, effector memory. A P value below 0.05 indicates a significant correlation.
